# Aerobic Exercise for Parkinson's Disease: A Systematic Review and Meta-Analysis of Randomized Controlled Trials

**DOI:** 10.1371/journal.pone.0100503

**Published:** 2014-07-01

**Authors:** Hai-Feng Shu, Tao Yang, Si-Xun Yu, Hai-Dong Huang, Ling-Li Jiang, Jian-Wen Gu, Yong-Qin Kuang

**Affiliations:** Department of Neurosurgery, General Hospital of the People's Liberation Army Chengdu Military Region, Chengdu, Sichuan, China; University of Toronto, Italy

## Abstract

**Background:**

Although some trials assessed the effectiveness of aerobic exercise for Parkinson's disease (PD), the role of aerobic exercise in the management of PD remained controversial.

**Objective:**

The purpose of this systematic review is to evaluate the evidence about whether aerobic exercise is effective for PD.

**Methods:**

Seven electronic databases, up to December 2013, were searched to identify relevant studies. Two reviewers independently extracted data and assessed methodological quality based on PEDro scale. Standardised mean difference (SMD) and 95% confidence intervals (CI) of random-effects model were calculated. And heterogeneity was assessed based on the *I^2^* statistic.

**Results:**

18 randomized controlled trials (RCTs) with 901 patients were eligible. The aggregated results suggested that aerobic exercise should show superior effects in improving motor actions (SMD, −0.57; 95% CI −0.94 to −0.19; p = 0.003), balance (SMD, 2.02; 95% CI 0.45 to 3.59; p = 0.01), and gait (SMD, 0.33; 95% CI 0.17 to 0.49; p<0.0001) in patients with PD, but not in quality of life (SMD, 0.11; 95% CI −0.23 to 0.46; p = 0.52). And there was no valid evidence on follow-up effects of aerobic exercise for PD.

**Conclusion:**

Aerobic exercise showed immediate beneficial effects in improving motor action, balance, and gait in patients with PD. However, given no evidence on follow-up effects, large-scale RCTs with long follow-up are warrant to confirm the current findings.

## Introduction

Parkinson's disease (PD) is a relatively progressive and neurodegenerative movement disorder that is characterized by many motor and non-motor symptoms such as resting tremor, bradykinesis, balance decrements, gait disruption, and reduced quality of life [1]. It is estimated that PD affects approximately 340,000 adults in the United States and this number would be probably doubled by the year of 2030 [2]. Although the causes of PD are still under investigation, its incidence obviously increases among people aged more than 50 years old [3]. In China, for example, PD prevalence is 1.70% in people aged more than 65 years old [4].

In recent years, aerobic exercise is widely used in assisting pharmacological treatments of PD. It may promote brain health by reducing inflammation, suppressing oxidative stress, and stabilizing calcium homeostasis [5]. Studies in healthy older rodents have shown that regular aerobic exercise triggered plasticity-related changes in the central nervous system, including synaptogenesis, enhanced glucose utilization, angiogenesis, and neurogenesis [6]. Other studies have shown that aerobic exercise, such as treadmill training, dancing, etc, may be beneficial in improving balance, gait, physical function, and quality of life in individuals with PD [7]–[9].

Some systematic reviews and meta-analyses supported that exercise therapies were effective in improving both motor and non-motor impairments of patients with PD [10], [11], but no review has addressed the specific effectiveness of aerobic exercise for PD. In the previous reviews, it is difficult to extract accurate information regarding the contribution of aerobic exercises in patients with PD because multiple exercise therapies were often involved.

Therefore, this systematic review aims to evaluate the evidence about whether aerobic exercise is effective for patients with PD. And we conducted meta-analyses of randomized controlled trials (RCTs) focusing specifically on balance, gait, and quality of life in patients with PD.

## Methods

### Search Strategy

The following electronic databases were searched from their inception to December 2013: PubMed, EMBASE, OVID-MEDLINE, Cochrane Library, CNKI (China Knowledge Resource Integrated Database), Weipu Database for Chinese Technical Periodicals, and Wan Fang Data. The following keywords were used in combinations: Parkinson, Parkinson's disease, Parkinsonism, exercise, physical activity, and physical therapy. Literature was also identified by citation tracking using reference lists from papers and internet searching. In order to include unpublished studies in our review, dissertations and trial registrations were also searched, and we contacted experts in this field. Two authors (HFS and TY) undertook the initial literature search and identified eligible studies. If it was unclear as to whether the study met the inclusion criteria, advice was sought from a third author and any disagreement was settled down by a consensus after discussion.

### Study Selection

The studies that met the following criteria were included: (1) RCTs of aerobic exercise for PD; (2) the target population was aged 20–85 years and confirmed diagnosis of PD; (3) the main intervention should be aerobic exercise and the exercise should be specifically suitable for the challenges and difficulties presented by PD; (4) the effect of aerobic exercise intervention was compared with any comparator, including other forms of exercise or physical activity; (5) the outcomes included at least one of the following: balance, gait, or health-related quality of life; (6) RCTs should contain available data for the meta-analysis; (7) the paper was available in either English or Chinese.

A study was excluded if: (1) the effect of a non-aerobic exercise intervention was evaluated (such as resistance training, behavioral interventions, music therapy, cueing strategies.); (2) the paper did not report outcomes for the first assessment period (cross-over studies only) so as to prevent any bias of carry over or order effects.

### Data Extraction

Two reviewers (HFS and SXY) independently extracted data onto predefined criteria in Table 1. We contacted primary authors when relevant information was not reported. Differences were settled by discussion with reference to the original article. For crossover studies, we considered the risk for carryover effects to be prohibitive, so we selected only the first phase of the study. First author, country, and year of the study were extracted as general study information. Population data, outcome assessments, interventions, and length of follow-up were taken to analyze the study characteristics.

**Table 1:** Characteristics of randomized controlled trials of aerobic exercise for PD.

First author, year, country	Hoehn and Yahr stage	Mean duration of PD (year)	Sample size, mean age (year)	Duration (week)	Follow-up (week)	Main outcome assessments	Experimental group intervention	Control group intervention
Thaut, 1996, US	NR	7	37, 71	3	—	Gait	Walking (30 min/9sessions)	1) Walking plus rhythmic auditory stimulation (30 min/9sessions); 2) Usual care
Miyai, 2000, Japan	2.5–3	4.2	10, 68	4	—	UPDRS, Gait	Body-weight-supported treadmill (45 min/12sessions)	Physical therapy (45 min/12sessions)
Miyai, 2002, Japan	2.5–3	4.3	20, 70	4	24	UPDRS, Gait	Body-weight-supported treadmill (45 min/12sessions)	Physical therapy (45 min/12sessions)
Protas, 2005, US	2–3	7.6	18, 72	8	—	Gait	Gait and step training (60 min/24sessions)	Usual care
Burini, 2006, Italy	2–3	11	26, 64	7	—	UPDRS, Gait, PDQ-39	Aerobic exercise (45 min/20sessions)	Qigong (50 min/20sessions)
Cakit, 2007, Turkey	2–3	5.6	31, 72	8	—	Gait, BBT	Treadmill training	Usual care
Fisher, 2008, US	1–2	1	30, 63	8	—	UPDRS, Gait	Body-weight-supported treadmill (45 min/24sessions)	1) Traditional physical therapy (45 min/24sessions) 2) Education (60 min/6sessions)
Hackney, 2008, US	1.5–3	7.1	33, 64	13	—	UPDRS, BBS, Gait	Tai Chi (60 min/20sessions)	No intervention
Frazzitta, 2009, Italy	3	13	40, 71	4	—	UPDRS, Gait	Treadmill training associated with auditory and visual cues (20 min/28sessions)	Auditory and visual cues
Hackney, 2009, US	1–3	7.3	48, 67	13	—	UPDRS, BBS, Gait	1) Tango; 2) Waltz/Foxtrot (60 min/20sessions)	No intervention
Sage, 2009, Canada	NR	3.5	36, 66	12	—	UPDRS, Gait	Aerobic exercise (30 min/36sessions)	1) Sensory attention focused exercise (40–60 min/30–34sessions); 2) Waiting list
Reuter, 2011,Germany	2–3	5.5	90, 63	24	—	UPDRS, Gait, PDQ-39	1) Nordic walking (70 min/72sessions); 2) Walking (70 min/72sessions)	Flexibility and relaxation (70 min/72sessions)
Canning, 2012,Australia	1–2	5.5	20, 62	6	6	UPDRS, Gait, PDQ-39	Home-based treadmill training (30–40 min/24sessions)	Usual care
Li, 2012, US	1–4	7	195, 69	24	—	UPDRS, Gait	Tai Chi (60 min/48sessions)	1)Stretching; 2)Resistance training (60 min/48sessions)
Picelli, 2012, Italy	3–4	7.5	34, 68	4	4	UPDRS, Gait, BBS	Robot-assisted gait training (40 min/12sessions)	Physical therapy
Schenkman, 2012, US	1–3	4.5	121, 64	64	—	UPDRS, FRT, PDQ-39	Aerobic exercise (45–60 min/320–448sessions)	1)Flexibility/balance/function exercise; 2) Home-based exercise (45–60 min/320–448sessions)
Shulman, 2013, US	1–3	6.2	67, 66	12	—	UPDRS, Gait	1) Higher-intensity treadmill (30 min/36sessions); 2) Lower-intensity treadmill (50 min/36sessions)	Stretching and resistance
Amano, 2013, US	2–3	8	45, 66	16	—	UPDRS, Gait	Tai Chi (60 min/32–48sessions)	1) Qigong (60 min/32–48sessions); 2) No exercise

PD: Parkinson's disease; NR: No reported; UPDRS: Unified Parkinson's Disease Rating Scale; PDQ39: Parkinson's Disease Questionnaire 39; BBT: Berg Balance Test; BBS: Berg Balance Scale; FRT: Functional Reach Test.

### Quality Assessment

The methodological quality of RCTs was assessed independently by two reviewers (SXY and HDH) with PEDro scale, which is based on the Delphi list and has been reported to have a fair-to-good reliability for RCTs of the physiotherapy in systematic reviews [12], [13]. The PEDro score ranged from 0 to 10 points. A cut point of 6 on the PEDro scale was used to indicate high-quality studies as this had been reported to be sufficient to determine high quality versus low quality in previous studies [12]. Disagreements were resolved by discussion between the reviewers, with the information of the primary author being sought if necessary. The PEDro scores were all settled down by consensus.

### Data Analysis

Meta-analysis was conducted with Cochrane Collaboration software (Review Manager Version 5.1). For continuous data, standardized mean difference (SMD) and 95% confidence intervals (CI) of random-effects model were calculated for all eligible trials. Heterogeneity across studies was tested based on the *I^2^* statistic, a quantitative measure of inconsistency across studies, and studies with *I^2^*<40% was considered to have low heterogeneity, *I^2^* of 40% to 75% was considered moderate heterogeneity, and *I^2^*>75% was considered high heterogeneity. Trials, including 2 similar intervention or control groups, had the groups combined with computational formula provided by the Cochrane handbook to create a single pair-wise comparison. Detailed subgroup analyses were conducted based on different outcomes and outcome measures.

## Results

### Study Selection

**Figure 1:**
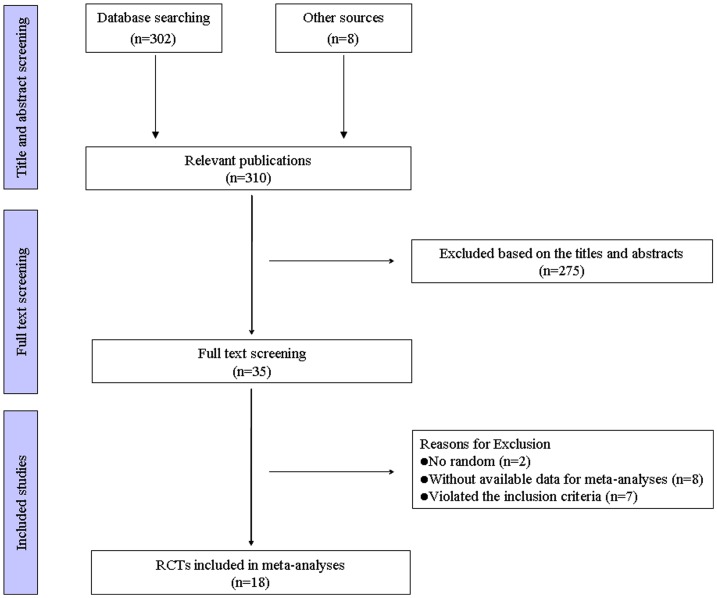
Flow chart for this meta-analysis. RCTs: randomized controlled trials.

Searching identified 310 records, of which 35 documents were retrieved from the screening of titles and abstracts. At last, 18 trials published between 1996 and 2013 were included in our meta-analysis [7]–[9], [14]–[28]. 17 literatures were eliminated for the reasons that 2 of them failed to randomize [29], [30], 8 without available data for the meta-analyses [31]–[38], and 7 violated the inclusion criteria [39]–[45]. Detailed selection process was showed in Figure 1. In course of document screening, no divergent views were found between the reviewers.

### Study Characteristics

#### Participants

There were 901 patients in the 18 eligible RCTs. Mean and standard deviation (SD) of age for all participants was 67±3.3 years, and the PD duration was 6.4±2.7 years. Most trials recruited participants with mild-to-moderate PD, including 14 with Hoehn and Yahr stage I to III [7], [8], [15]–[22], [24], [26]–[28] and 2 with Hoehn and Yahr stage I to IV [9], [25].

#### Interventions

All the eligible literatures reported aerobic exercise interventions including treadmill training, Tai Chi, walking, dancing, etc. The interventions in control group were various, such as no intervention, usual care, stretching, resistance exercises, physical therapy, and other exercise. The intervention time spanned from 3 weeks to 16 months. Detailed characteristics of the included trials were summarized in Table 1.

### Methodological Quality

The quality of the included studies was summarized in Table 2. The total scores for the methodological quality ranged from 4 to 8 points. No studies reported subjects-blinding and therapists-blinding, which were the common failing for the non-pharmacological clinical trials. However, most of them (78%) performed assessors-blinding [7]–[9], [17]–[21], [23]–[28]. Although all trials adopted random assignment of patients, only 4 used adequate method of allocation concealment [8], [18], [20], [26]. The expulsion of 7 studies was definitely higher than 15% [7], [16], [18], [19], [21], [26], [27]. As for the intention-to-treat analysis, 9 trials were failed for cancelling the dropout data in the last results [7], [16], [18], [19], [21], [23], [25]–[27]. For the remaining items on PEDro scale, the eligible studies showed a high methodological quality.

**Table 2:** PEDro scale of quality for eligible randomized controlled trials.

Study	Eligibility criteria	Random allocation	Concealed allocation	Similar at baseline	Subjects blinded	Therapists blinded	Assessors blinded	<15% dropouts	Intention-to-treat analysis	Between-group comparisons	Point measures and variability data	Total
Thaut, 1996 US	1	1	0	1	0	0	0	1	1	1	1	6
Miyai, 2000, Japan	1	1	0	1	0	0	0	1	1	1	1	6
Miyai, 2002, Japan	1	1	0	1	0	0	0	0	0	1	1	4
Protasa, 2005, US	1	1	0	1	0	0	1	1	1	1	1	7
Burini, 2006, Italy	1	1	1	1	0	0	1	0	0	1	1	6
Cakit, 2007, Turkey	1	1	0	1	0	0	1	0	0	1	1	5
Fisher, 2008, US	1	1	1	1	0	0	1	1	1	1	1	8
Hackney, 2008, US	1	1	0	1	0	0	1	0	0	1	1	5
Frazzitta, 2009, Italy	1	1	0	1	0	0	0	1	1	1	1	6
Hackney, 2009, US	1	1	0	1	0	0	1	0	0	1	1	5
Sage, 2009, Canada	1	1	0	1	0	0	1	1	0	1	1	6
Reuter, 2011,Germany	1	1	0	1	0	0	1	1	1	1	1	7
Canning, 2012,Australia	1	1	1	1	0	0	1	1	1	1	1	8
Li, 2012, US	1	1	0	1	0	0	1	1	1	1	1	7
Picelli, 2012, Italy	1	1	0	1	0	0	1	1	0	1	1	6
Schenkman, 2012, US	1	1	1	1	0	0	1	0	0	1	1	6
Shulman, 2013, US	1	1	0	1	0	0	1	0	0	1	1	5
Amano, 2013, US	1	1	0	1	0	0	1	1	1	1	1	7

Criteria (2–11) were used to calculate the total PEDro score. Each criterion was scored as either 1 or 0 according to whether the criteria was met or not, respectively.

### Quantitative Data Synthesis

#### Unified Parkinson's disease rating scale (UPDRS)

**Figure 2:**
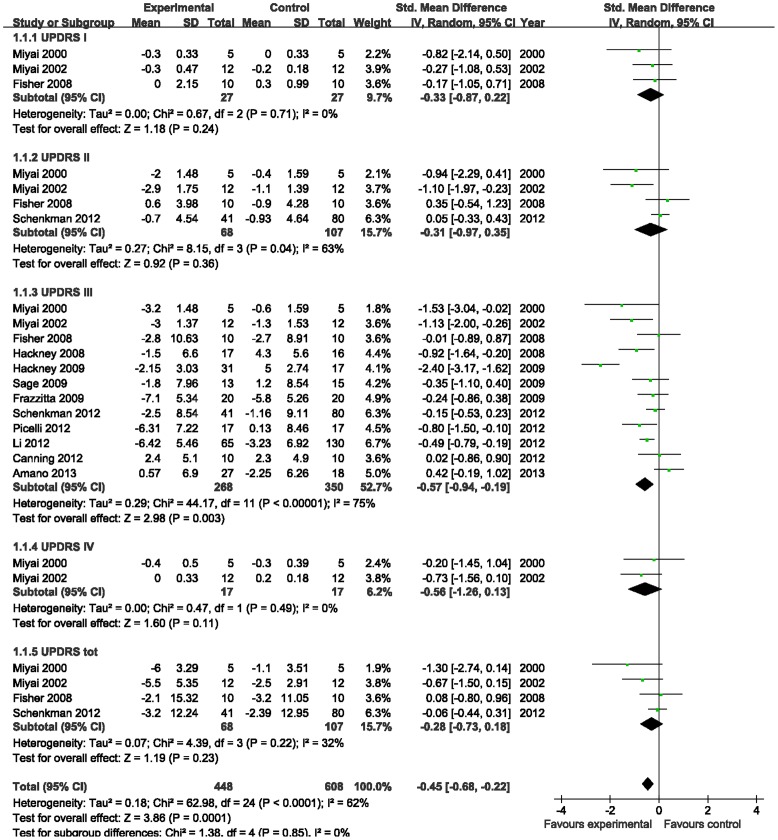
Forest plot showing the effect of aerobic exercise on unified Parkinson's disease rating scale (UPDRS).

UPDRS, as the most common marker in the clinical study of PD, was employed in most eligible RCTs. The aggregated result showed a statistically significant benefit in favor of aerobic exercise for PD in UPDRS III (SMD, −0.57; 95% CI −0.94 to −0.19; p = 0.003; Figure 2) [7]–[9], [15], [16], [20]–[23], [25], [26], [28]. But it was not associated with significant improvements in UPDRS I (SMD, −0.33; 95% CI −0.87 to 0.22; p = 0.24; Figure 2) [15], [16], [20], UPDRS II (SMD, −0.31; 95% CI −0.97 to 0.35; p = 0.36; Figure 2) [15], [16], [20], [26], UPDRS IV (SMD, −0.56; 95% CI −1.26 to 0.13; p = 0.11; Figure 2) [15], [16], nor UPDRS tot (SMD, −0.28; 95% CI −0.73 to 0.18; p = 0.23; Figure 2) [15], [16], [20], [26]. This suggested that aerobic exercise could positively improve motor actions in patients with PD.

#### Balance

**Figure 3:**
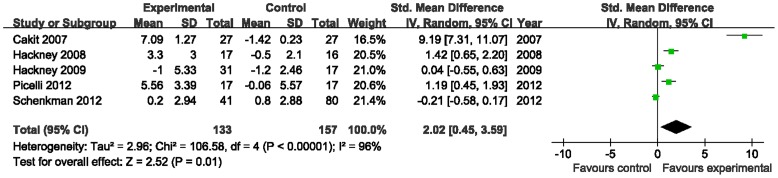
Forest plot showing the effect of aerobic exercise on balance in patients with Parkinson's disease.

5 studies assessed equilibrium function of patients with PD. Nearly half of trials showed favorable effects of aerobic exercise in improving balance in patients with PD, and the aggregated result also supported it (SMD, 2.02; 95% CI 0.45 to 3.59; p = 0.01; Figure 3) [7], [19], [21], [25], [26].

#### Gait

**Figure 4:**
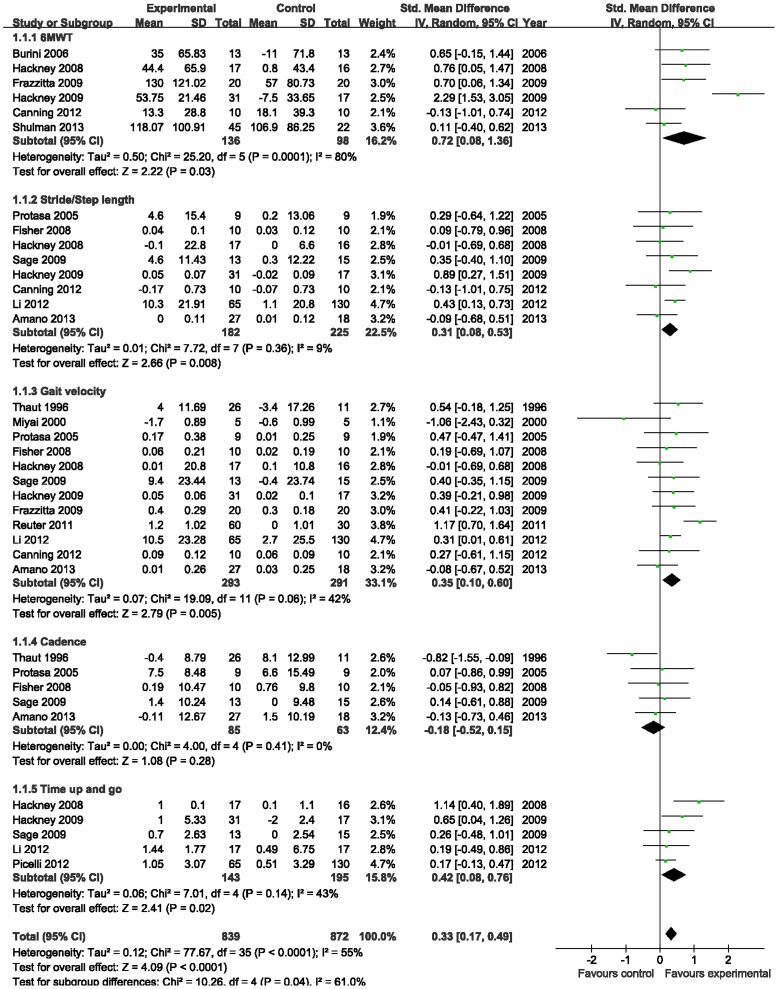
Forest plot showing the effect of aerobic exercise on gait in patients with Parkinson's disease.

Aerobic exercise showed superior effects in improving gait in patients with PD (SMD, 0.33; 95% CI 0.17 to 0.49; p<0.0001; Figure 4). 6-minute walking test, stride/step length, gait velocity, cadence, and time up and go were analyzed in eligible studies. The aggregated results suggested that aerobic exercise should show significant effects compared with control therapies in 6-minute walking test (SMD, 0.72; 95% CI 0.08 to 1.36; p = 0.03; Figure 4) [7], [8], [21], [22], [27], stride/step length (SMD, 0.31; 95% CI 0.08 to 0.53; p = 0.008; Figure 4) [7]–[9], [17], [21]–[23], [28], gait velocity (SMD, 0.35; 95% CI 0.10 to 0.60; p = 0.005; Figure 4) [7]–[9], [14], [15], [17], [20]–[24], [28], and time up and go (SMD, 0.42; 95% CI 0.08 to 0.76; p = 0.02; Figure 4) [7], [9], [21], [23], [25]. However, none of the trials indicated the evidence in favor of aerobic exercise for PD in the assessment of the cadence (SMD, −0.18; 95% CI −0.52 to 0.15; p = 0.28; Figure 4) [14], [17], [20], [23], [28].

#### Quality of life

**Figure 5:**
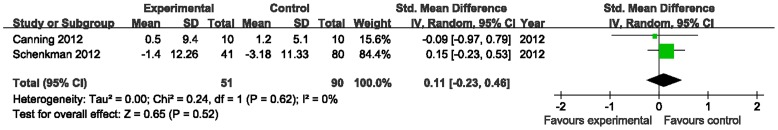
Forest plot showing the effect of aerobic exercise on quality of life in patients with Parkinson's disease.

Four trials reported beneficial effects of aerobic exercise for PD in the quality of life, but there was no difference between aerobic exercise and control therapies [8], [18], [24], [26]. And the synthetical effect size did not either show superior effects of aerobic exercise (SMD, 0.11; 95% CI −0.23 to 0.46; p = 0.52; Figure 5) [8], [26].

### Follow-up Effect

3 trials reported the follow-up effects of aerobic exercise for PD. The follow-up duration ranged from 4 weeks to 24 weeks. 1 study showed persistency effects of aerobic exercise in the number of steps for the 10-m walk [16], 1 in quality of life [8], and 1 in balance, gait, and motor action [25].

### Adverse Events

Only two studies reported non-serious adverse events during the aerobic exercise training period. Two patients experienced hypotension in hot weather, four fell due to obstacles, five twisted ankles during cross-country walking, and one complained of pain in Reuter's study [24]. The other study reported one non-injurious fall and two complaints of soreness or pain during aerobic exercise intervention [26].

## Discussion

This is the first systematic review to evaluate the effectiveness of aerobic exercise for PD. Our meta-analyses suggested that aerobic exercise significantly improve motor action, balance, and gait including gait velocity, stride/step length, and walking ability in patients with PD. Currently, there was no sufficient evidence to support or refute the value of aerobic exercise in improving quality of life in patients with PD compared with other therapies. And there was no valid evidence on follow-up effects of aerobic exercise for PD.

In our systematic review, most eligible trials showed moderate methodological quality based on PEDro score, which suggested that our findings were believable. We analyzed the 18 RCTs of aerobic exercise, including treadmill training, dancing, walking, and Tai Chi for PD conditions. Our aggregated results supported that aerobic exercise showed superior effects in improving motor action, balance, and gait in patients with PD. It was similar to related systematic reviews. Herman's systematic review suggested that treadmill training should play an important role in improving gait and mobility in patients with PD [46]. But it was only a qualitative review, and any strictly qualitative approach may be problematic since it can be more subjective than meta-analyses. Mehrholz's review also concluded that treadmill training was likely to improve gait hypokinesia and showed better safety [47]. Comparing with these reviews, larger new eligible RCTs (the last searching December 2013), more electronic databases (especially including 3 Chinese databases), and detailed subgroup meta-analyses (motor action, balance, gait and quality of life) strengthened our confidence in our systematic review.

In our review, two parts of analyses (UPDRS II and quality of life) came to the same conclusion that there was no sufficient evidence to support or refute the value of aerobic exercise in improving quality of life in patients with PD compared with other therapies. Some related systematic reviews drew a different conclusion that the evidence supported exercise as being beneficial with regards to health-related quality of life for patients with PD [48], [49], but the evidence was gained through the utilization of various exercise therapies. The reviews, focusing on aerobic exercise for PD, only suggested that treadmill training potentially improve quality of life in patients with PD [46], [47]. Modestly, our review included meta-analyses with larger data, but more trials were warranted to prove it.

PD is a complex disease that can compromise physical performance. Depending on the symptom severity, PD can present many obstacles to traditional exercise programming. Tough movement can be difficult for PD patients to perform. Recently, more studies have reported that intensive exercise achieved optimal results in the rehabilitation of patients with PD [50]. In these studies, intensity of exercise interventions depends on frequency and duration of exercises, number of repetitions, and complexity of exercises. A treatment is generally considered intensive when involving 2 to 4 hours of exercises per week, for 6 to 14 weeks. And larger studies reported the effect of intensive exercise in improving cell proliferation and neuronal differentiation [50]–[52]. In our review, almost 80% of aerobic exercises are intensive exercise. And the intensive aerobic exercise showed superior effects in improving motor action, balance, and gait of patients with PD.

Based on maximal heart rate or metabolic equivalents, the recent studies have showed that lower-intensity treadmill training yields more improvements in gait velocity than higher-intensity treadmill training for PD patients [27]. But Fisher's study led to different conclusion in gait of early PD undergoing high-intensity body weight-supported treadmill training [20]. The different conclusions may be rooted in the variation in types of treadmill training, duration and amount of exercises, patient characteristics, and main outcome measures. However, the physical activity guidelines reported by the U.S. Department of Health and Human Services suggested that moderate intensity physical activity could generate multiple health benefits [53]. Considering the disease characteristics and safety of exercise interventions, moderate or light intensity exercises should be considered beneficial and adoptive for individuals with PD [54]–[56]. In our review, the Hohen and Yahr stage of participants mainly arranged from I to III. So the exploration of these RCTs suggested that low to moderate intensity aerobic exercise, such as treadmill training, Tai Chi, dancing, etc, would benefit mild to moderate staged PD patients. However, the evidence is not conclusive. Future research should further investigate intensity level of aerobic exercise to check and monitor its effectiveness based on maximal heart rate or metabolic equivalents.

There were some limitations in our review. The large span of the durations (from 3 weeks to 64 weeks) in aerobic exercise interventions could influence our analysis. So it is difficult to conduct subgroup analyses on the different durations of aerobic exercise and determine the optimal size of aerobic exercise for PD. And there was insufficient data for the follow-up effect of aerobic exercise for PD, which is important for final decision of the clinicians. In addition, we could not get rid of the publication bias due to retrieval of documents in English and Chinese databases only.

## Conclusions

This systematic review shows the positive evidence that aerobic exercise has immediate beneficial effects in improving motor action, balance, and gait in patients with PD. However, this is not sufficient to reach any definitive conclusion because there are very few studies with a follow-up evaluation. Large-scale RCTs with long follow-up are warrant to confirm the current findings of aerobic exercise for PD.

## Supporting Information

Checklist S1 PRISMA Checklist.
(DOC)
